# Indian Herb-Derived Phytoconstituent-Based Antiviral, Antimicrobial and Antifungal Formulation: An Oral Rinse Candidate for Oral Hygiene and the Potential Prevention of COVID-19 Outbreaks

**DOI:** 10.3390/pathogens10091130

**Published:** 2021-09-02

**Authors:** Shashwat Sharad, Suman Kapur

**Affiliations:** 1 Center for Prostate Disease Research, John P. Murtha Cancer Center Research Program, Department of Surgery, F. Edward Hebert School of Medicine, Uniformed Services University of the Health Sciences and Walter Reed National Military Medical Center, Bethesda, MD 20817, USA; 2 Department of Biological Sciences, Birla Institute of Technology and Science, Pilani, Hyderabad Campus, Hyderabad 500078, India

**Keywords:** antiviral agents, coronavirus, COVID-19, SARS-CoV-2, natural herb, phytoconstituents, horizontal transmission, shedding, mouthwash, gargle

## Abstract

Outbreaks of emerging infectious diseases continue to challenge human health. Novel severe acute respiratory syndrome coronavirus-2 (SARS-CoV-2) has triggered a global coronavirus pandemic, known as COVID-19. Multiple variants of SARS-CoV-2 virus are circulating, thus raising questions with respect to the effectiveness of different lines of treatment, such as vaccines and antiviral drugs. To find the appropriate prevention/treatment, 21 plant-based ingredients (Glycyrrhizin, Withanone, Aloe-emodin, Rhein, Emodin, Chrysophanol, Physcion, Kaempferol, Progallin A, Gallic acid, Naringin, Quercetin, Luteolin, and Apigenin) having antiviral, antibacterial and antifungal properties were identified. We pseudo-typed SARS-CoV-2 on a lentiviral vector plasmid and tested the impact of five different herbal formulations in mammalian HEK293T cells. Viral inactivation assay showed that the natural extracts in a herb-derived phytoconstituent-based formulation, BITS-003, comprising *Bacopa monnieri*, *Glycyerrhiza glabra*, *Asparagus racemosus-wild*, and *Nigella sativa* had strong virucidal properties, inactivating enveloped viruses from 2log10 (or 99%) to >4log10 (or 99.99%). Moreover, bacterial and yeast cells treated with BITS-003 displayed reduced growth. Topical use of the formulation as a mouthwash/gargle could be effective in reducing symptoms of respiratory viral infections, with the potential to decrease the viral load in the buccal/oral cavity. This may inhibit the coronavirus spreading to the lungs of infected persons and at the same time may reduce the risk of viral transmission to other susceptible persons through micro-droplets originating from the oral cavity of the infected person.

## 1. Introduction

The incidence of emerging and re-emerging zoonotic diseases is increasing globally. Influenza viruses, which circulate in all parts of the world, cause seasonal epidemics [1]. Coronaviruses are a large family of positive-stranded RNA viruses that cause minor and major infectious diseases in mammals, including humans. For decades, common human coronaviruses (HCoVs) have circulated in the human population without any significant mortality [2,3,4,5,6,7]. However, in less than 20 years, three new HCoVs have emerged, causing severe respiratory syndromes and significant mortality [8,9,10,11,12,13,14,15,16]. Several viral infections have created serious threats in the last two decades, claiming thousands of human lives. Severe Acute Respiratory Syndrome Coronavirus (SARS-CoV) first emerged 17 years ago [9]. In December 2019, a novel coronavirus, defined by the World Health Organization (WHO) in January 2020 as severe acute respiratory syndrome coronavirus 2 (SARS-CoV-2), crossed species barriers to infect humans [17,18,19,20]. This novel SARS-CoV-2 is the etiologic agent of the outbreak of pneumonia in Wuhan, China, in late 2019, known as coronavirus disease 2019 (COVID-19), and it spread to all the countries across the globe. The virus was found to be a member of the beta coronavirus family, in the same species as SARS-CoV and SARS-related bat coronaviruses [21,22]. Unlike its predecessors, SARS-CoV-2 spread rapidly, reaching pandemic levels within two months; at the time of writing this manuscript, the virus has infected over 176 million (176,895,871) people, with more than 3.8 million (3,822,394) deaths worldwide [23]. Patterns of spread indicate that SARS-CoV-2 is transmitted from person to person, and several mutant strains of the virus have emerged in last 18 months [22,24,25]. The major method of transmission of SARS-CoV-2 is through aerosolized respiratory droplets. Some of the most common symptoms of COVID-19, such as coughing and sneezing, are associated with the formation of aerosols [15,19]. Persons infected but showing only mild or no symptoms (asymptomatic infection) can readily spread the virus through aerosols [15,19,26,27]. Virus on surfaces (fomites) can remain viable for hours or even days and may represent an important secondary mode of transmission [9,15,26,27,28].

While there is a potential for other mechanisms, aerosolization and fomites are considered the most probable means of transmission and spread. The nasal and oral cavities are the major entry points for SARS-CoV-2 [29]. The spike protein, one of the structural outer proteins of coronaviruses, mediates virus binding and entry into human cells [30]. Initial characterization of the SARS-CoV-2 spike protein indicate that it binds the same receptor, angiotensin-converting enzyme 2 (ACE2), as SARS-CoV, which is expressed in both the upper and lower human respiratory tracts [30,31].

Currently, specific therapies for the early containment and prevention of transmission and spread of SARS-CoV-2 are lacking. SARS-CoV-2 can be transmitted via saliva; when an infected person sneezes, coughs, breaths or converses, saliva droplets containing microorganisms are produced, posing the risk of transmission [32]. Although vaccines have been developed, their efficacy against emerging mutant strains remains an open question. Moreover, the long-term protection offered by immunization is yet to be ascertained. The SARS-Cov-2 viral epidemic has raised the issue of developing effective antiviral agents at the earliest stage to prevent further losses. At present, although a series of candidate drugs have been reported to ameliorate respiratory symptoms, there are still no clinically approved or recommended antiviral drugs specified for SARS. Bacterial/fungal co-infection of the respiratory tract in patients with coronavirus has also been reported. Mouthwashes and gargles are used as an adjunct therapy in oral health. Considering mouthwash and gargle as oral rinse candidate agents can decrease the viral load (including SARS-CoV-2), microbial load and fungal load and could be of interest in the fight against COVID-19. [33,34,35]. Natural products have always played a crucial role in the drug development process for various diseases; similarly, such agents have been screened for combatting emergent new variants of coronaviruses. Inhibition of viral and microbial (bacteria, fungus, germs) replication is often considered as a general mechanism for the antiviral and antimicrobial activity of most of these natural products. Studies have shown that some natural products can interact with key viral elements, such as the viral envelope and embedded proteins, which are associated with infectivity and virulence [36]. In light of this, extracts from a number of medicinal plants and multiple formulations thereof were tested for their ability to inhibit viral, bacterial and fungal infectivity. Several formulations comprising four to fourteen extracts were tested for antiviral activity using a lentiviral system and cultured HEK 293TN cells, and the antibacterial and antifungal properties of formulation BITS-003 were also tested.

## 2. Materials and Methods

### 2.1. Mouthwash Formulations and Sources of Compounds Tested 

Several natural product extracts were procured from Good Manufacturing Practices (GMP) and International Organization for Standardization (ISO) certified manufacturers in India. The herbal extracts were prepared as per the standard guidelines described in the Indian Systems of Medicine (ISM) pharmacopeia [37]. The 17 extracts of medicinal plants with known antiviral, antibacterial and antifungal activity were obtained (Table 1). The aqua-ethanolic extracts were characterized for their polyphenol, flavonoid and saponin contents. Selected plant extracts were mixed in different proportions and double diluted with sterile phosphate buffer saline (PBS). The five different formulations (BITS-001 to BITS-005) were prepared by mixing selected plant extracts in different proportions, and an appropriate volume of each component was mixed in a glass vessel with constant stirring and filtered through Whatman filter paper 1 before further use. All the plant extracts selected and tested in this study were completely free of peroxide, betadine, providone, iodine, salicylate, menthol, thymol, eucalyptol and any other known antiseptic drugs. The compounds present in various formulations are Glycyrrhizin, Withanone, Aloe-emodin, Rhein, Emodin, Chrysophanol, Physcion, Kaempferol, Progallin A, Gallic acid, Naringin, Quercetin, Luteolin and Apigenin, with reported antiviral, antibacterial and antifungal activity.

### 2.2. Determination of Total Polyphenol Content

The estimation of total polyphenols was carried out using the Folin-Ciocalteu (F-C) colorimetric method. The chemical reaction between the polyphenol and the F-C reagent yields a blue color complex, and the color is read at 760 nm. Briefly, 50 µL plant extract was homogenously mixed with 50 µL of F-C reagent, and the reaction mixture was incubated at room temperature for 2 min. The reaction was stopped after the incubation period by adding 500 µL of 5% sodium carbonate (*w*/*v*). Finally, 400 µL water was added to make up the volume to 1 mL, and the reaction mixture was subsequently heated at 45 °C for 30 min, followed by cooling at room temperature. A gallic acid standard curve was prepared for concentrations ranging from 0–300 µg/mL (r^2^ = 0.998). All experiments were done in triplicate, and the results were expressed as mean ± SD.

### 2.3. Determination of Total Flavonoid Content

Total flavonoid content in the herbal extract was measured using the Aluminum chloride method (15). Briefly, 50 μL of herbal extract was homogenously mixed with 100 μL of 2% AlCl_3_, and the reaction mixture was incubated in darkness for 1 hour at room temperature. Absorbance of the reaction mixture was measured at 405 nm in an ELISA reader (BioTek, Elx 800, Winooski, VT, USA). A standard calibration curve of Rutin was plotted for concentrations ranging from 0 to 1000 μg/ml with a linear fit (r^2^ = 0.9896). All samples were analyzed in triplicate, and the results were expressed as mean ± SD.

### 2.4. Estimation of Total Saponin Content 

Vanillin-sulfuric acid assay was used to measure the total saponin content of the herbal extracts. In brief, 10 μL of each herbal extract was homogenously mixed with 20 μL of vanillin reagent (8%, *w*/*v* in 99.9% ethanol) followed by addition of 200 μL of 72% (*v*/*v*) sulfuric acid to each tube. The reaction mixture was vortexed and heated in a water bath at 60 °C for 10 min. After completion of the reaction, the final reaction mixture was cooled to room temperature. An appropriate blank (tube without saponin) was run, and a standard calibration curve at various concentrations of saponin (10–80 μg/mL) was obtained with a linear fit (r^2^ = 0.9916). The total saponin content of all herbal extracts was expressed as saponin equivalents (µg/ml). All samples were analyzed in triplicate, and the results were expressed as mean ± SD.

### 2.5. Cell Maintenance 

The HEK293 cell lines were maintained in their respective media, supplemented with 10% FBS and without antibiotic, with a media change every 2–3 days until 70% confluency was attained. Sub-culturing of the cells was carried out using 0.25% trypsin-EDTA solution. Quality checks were performed on the cells using Mycoplasma testing.

### 2.6. Cytotoxicity Assay for Screening Various Formulations

For determining the maximum non-cytotoxic concentration of different natural extracts (60% ethanol), an initial cytotoxicity assay was conducted for HEK293 cells. The optimization of cell culture parameters was carried out before screening the test compounds for their effect on the cell viability. The HEK293 cells were plated at two different densities, and the ethanol tolerance (0.01–5%) and ethanol exposure time (24 h, 48 h, 72 h) were tested as a control. The luminescence detection was done using the instrument, Molecular Devices Flex Station 3/ SPARK 20M (Tecan, Mennendorf, Switzerland). For the cytotoxicity screening, the formulations were diluted at 1:30, 1:60, 1:120 and 1:240 in a 96-well plate format to assess compound safety at specific concentrations for dispensing of a drug sample. Media alone (without cells) + Cell Titer Glo reagent was used as background control, and media alone in the presence of a vehicle control served as a negative control for maximum viability. The cell viability number and optimum ethanol concentration was determined using a homogenous “Cell Titer Glo” assay method (Promega Cat. No. G7573), whereby luminescence output obtained in relative light units (RLUs) was plotted against the time point or seeding density. Based on the optimization of cell culture parameters (seeding density, ethanol tolerance and exposure), the screening of the test compounds was carried out. 

### 2.7. Cloning and Generation of Expression Lentivectors 

The lentiviral shRNA expression system from biosciences (catalogue no# s SI500A-1) was used for pSIH-H1 shRNA cloning and generating the expression lentivectors (pSIH1-H1-Puro, pSIH1-H1-H2Kk and pSIH1-H1-copGFP Vectors) as per the manufactures’ protocol. The pSIH-H1 vectors are designed to express a single-stranded shRNA sequence with a fold-back stem-loop structure (also known as a “hairpin”) from a RNA polymerase III H1 promoter. For DNA scale-up of pLenti-GFP lentiviral vector, the pSIH1-H1 (Codes for GFP, System Biosciences) was transformed into DH5α competent cells, and purified Lentiviral vector was obtained using Qiagen maxi column kit (QIAGEN Inc, Valencia, CA, USA).

### 2.8. Generation of Lentiviral Particles

HEK293TN cells were plated. After 24 h, transient transfection of the HEK293TN cells with the lentivirus packaging vector (pPACKH1-System Bioscience) and the pSIH1-H1 lentiviral transfer vector was carried out. The lentivirus particles were collected after 72 h, and the virus was concentrated using the LentiX concentrator (Clontech, Taipei, Taiwan). The lentiviral particles were titrated using HEK293TN cells in a 24-well format and finally aliquoted and stored at −80 °C. If Lenti-GFP virus titer was found to be lower than expected (0.5–1 × 10^6^/mL), virus preparation was re-initiated with a fresh batch of HEK293FN cells. 

### 2.9. Viral Inactivation Assay and Infection Inhibition Assay

The 24-well plates were pre-coated with Poly-L-lysine (1:10 dilution) in 1X PBS for 1 h at room temperature. A total of 40,000 HEK293TN cells in 480 µL of complete medium were plated in a single well of polylysine-coated 24-well plate. A total of 60 µL of the lentiviral particles in OptiMEM medium was incubated with 2 µL of extract for 10 min at room temperature. Spent medium was removed from the well and replaced with 460 µL of polybrene (8 µg/µL) containing complete medium. A total of 20 µL of virus, inactivated with extract, was added to the wells. The viral inactivation assay was conducted using 5 formulations. The above-indicated concentration (2 µL extract in 60 µL of lentivirus for viral inactivation assay) was used for all of the 5 extracts (BITS-001–BITS-005). The viral inactivation assay was conducted in triplicate for each extract. 

### 2.10. Fluorescence-Activated Cell Sorting (FACS) and Data Analysis

The HEK293TN cells were transduced with the inactivated lentiviral particles, pSIH1-H1, in the presence of 1X TransDux (System Biosciences, Palo Alto, CA, USA)), and pre-treated monolayer cells were transduced with viral particles (to achieve a minimum 4–5% transduction efficiency). After 72 h of incubation at 37 °C in the CO_2_ incubator (5% CO_2_), green fluorescence protein (GFP) expression was quantified using FACS (Fluorescence Activated Cell Sorting) analysis to score the percentage of inactivation of viral particles and viral infection inhibition using Novocyte Advanteon Flow Cytometer System (NovoExpress Software, Agilent, Santa Clara, CA, USA). Bright field and fluorescent images were taken for each condition, using a Nikon ECLIPSE TS2 microscope (NIS Elements version 4.0, 64-bit images) at 10× magnification. The calculation of the average absorbance of the media plus Cell Titer Glo reagent without cells from triplicates was used as the “average background signal” in control wells. The average background signal was subtracted from all other absorbance values (raw data) to obtain background adjusted values. The normalization of “background adjusted values” was done to the average value of vehicle-treated cells set to be equivalent to 100% viability, known as “normalized viability values”. The bar graph plotted with the “normalized viability values” represent each formulation tested. Each graph corresponding to the individual formulation tested represents mean +/− SEM of three replicates. 

### 2.11. Estimation of Antibacterial Activity of the Herb-Based Formulation 

For evaluation of the antibacterial activity of the novel herb-based formulation number BITS-003, the formulation was diluted to a final concentration of 1:20, 1:40 and 1:80 for each constituent, as shown in Figure 3. In a representative experiment, 2.5 µL, 5 µL and 10 µL of constituents were mixed with 197.5 µL, 195 µL and 190 µL of 10^3^ bacterial cells, respectively, and incubated for 20 min at 37 °C. Finally, 50 µL of the said incubated mix was gently poured on an agar plate and incubated for 18 h at 37 °C for evaluation of antibacterial activity of the constituents and the herb-based formulation. The next day, bacterial growth was monitored as the number of colonies obtained in the control as well as the treated bacterial cells.

### 2.12. Estimation of Antifungal Activity of the Herb-Based Formulation

For evaluation of antifungal activity, the novel herb-based formulation, BITS-003, was diluted to the final concentration of 1:20, 1:40 and 1:80, as described in Figure 4. In one of the representative experiments 2.5 µL, 5 µL and 10 µL of the herb-based formulation was mixed with 197.5 µL, 195 µL and 190 µL of 10^3^ yeast cells and incubated for 20 min at 37 °C. Finally, 50 µL of the said incubated mix was gently poured on yeast extract peptone dextrose agar (YEPD) and incubated for ~48 h at 30 °C for evaluation of antifungal activity of the herb-based formulation. After ~48 h, the fungal growth was monitored in the control and as well as the treated fungal cells.

## 3. Results

### 3.1. Development of Indian Herb-Derived Phytoconstituent-Based Antiviral Formulation

The 17 medicinal plant extracts with known antiviral, antibacterial and antifungal activity were used to prepare five different formulations: BITS-001 to BITS-005 (Table 1). The selected plant extracts were mixed as described above. The final alcohol concentration in each formulation was 2.5% (as working strength was 1 to 30 times diluted). The formulation used Food Safety and Standards Authority of India (FSSAI)-approved natural products at the concentration specified in the nutraceutical’s guidelines. 

The RNA virus molecule is held inside a fatty envelope containing the outer S protein and two other proteins. The advisory of frequent hand sanitization is based on disrupting this fat layer to disable the virus. Saponin-rich components, used in alcohol-free mouthwash in our formulation, will disrupt the lipid envelope of the virus growing in the oral cavity of the infected person, reducing the viral load in the mouth (and thus reducing the likelihood of further spread to airway passages and the lungs) and also the propensity to shed the active virus in respiratory droplets/micro-droplets.

S glycoprotein has high content of mannose and manan-glycans. Some of the natural products in the formulations contain mannose and mannan glycan binders, which bind the S protein on the surface of the virus and thus inhibit further interaction of the virus with new cells in the infected person and also in the new host [34]. A large number of glycosylated S proteins cover the surface of SARS-CoV-2 and bind to the host cell receptor angiotensin-converting enzyme 2 (ACE2), mediating viral cell entry. The interaction with manan-glycans and the action of saponins have different mechanisms of action. The manan-glycans can block the interaction of the spike protein with ACE2 receptors. Through interaction with the glycation units on the spike protein, the saponin acts as a bio-surfactant, which disrupts the lipid envelope of enveloped viruses, leading to destruction of their ability to enter a new host cell. 

Thus, sanitizing the mouth with solutions (mouthwash) rich in saponins and mannose-binding molecules would benefit the actively infected person and also stop further transmission from person to person.

### 3.2. Phytoconstituents of the Natural Extracts and Herb-Based Formulation BITS-003

**Figure 1:**
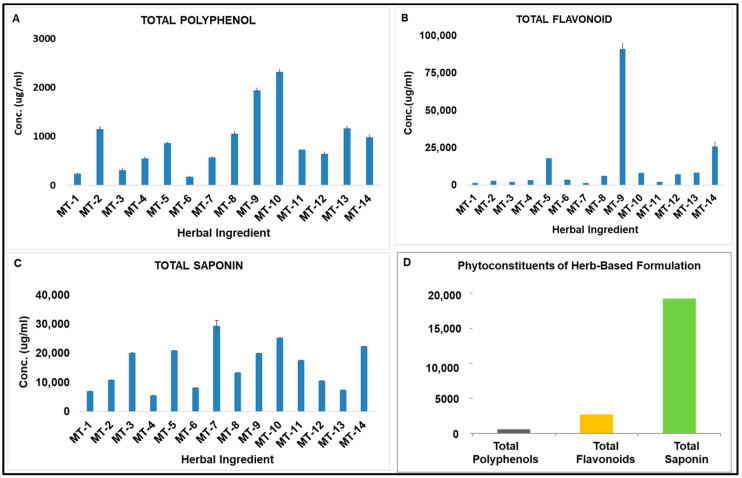
The phytoconstituents of 14 out of 17 selected herbal ingredients used in the preparation of oral herbal mouthwash. (**A**) Total Polyphenols, (**B**) Total Flavonoids (**C**) Total Saponins. MT-1: *Oryza Sativa*; MT-2: *Phaseolus Munga L*; MT-3: *Asparagus racemosus*; MT-4: *Solanum* *tuberosum*; MT-5: *Glycyrrhiza glabra*; MT-6: *Withania somnifera*; MT-7: *Zingiber officinale*; MT-8: *Bacopa monnieri*; MT-9: *Rheum palmatum*; MT-10: *Rosmarinus officinalis*; MT-11: *Capsicum annuum*; MT-12: *Colocasia antiquorum Schott*; MT-13: *Trigonella foenum graceum* and MT-14: *Nigella Sativa.* (**D**) Phytoconstituents of herb-based formulation BITS-003. Y-axis represents concentration of the phytoconstituent in microgram/ml. Note: Extracts of *Ananas Comosus, Morus nigra and Utica dioica* could not be tested due to either background color of the extract or interference with reactants and subsequent precipitation.

The phytoconstituents, polyphenols, flavonoids and saponins were measured in the herb-based formulation BITS-003. The concentration of total polyphenols was 643.63 µg/mL; total flavonoids ≥ 2500 µg/mL; total saponins ≥ 18,000 µg/mL (Figure 1). The constituents of all the formulations (BITS 001, 002, 003, 004 and 005) are shown in Table 2. The exact concentration of each of the three classes (total polyphenol, total saponin and total flavonoid content) of compounds in all five formulations are shown in Table 2. BITS-003 was primarily chosen for the high concentration of the bio-surfactant, namely saponins.

### 3.3. Determination of Maximum Non-Cytotoxic Concentration of Natural Extract Formulations in HEK293TN Cells

**Figure 2:**
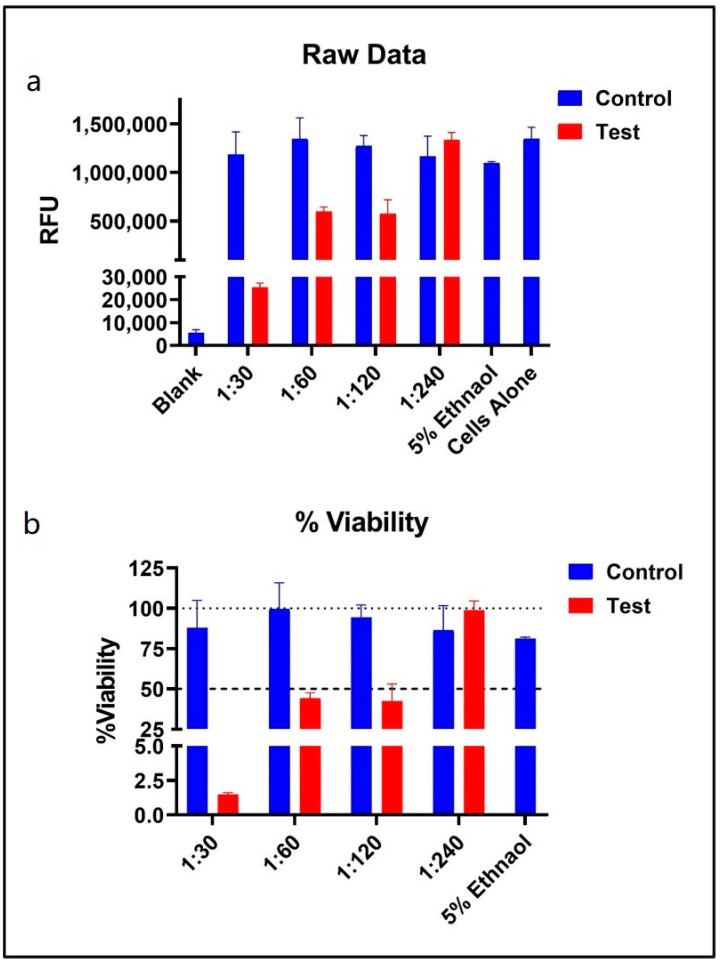
Representation of maximum non-cytotoxic concentration of BIT-003 natural extract formulation in HEK293TN cells. (**a**) Raw data analysis of the relative light units (RLU). (**b**) Cell viability analysis.

The maximum non-cytotoxic concentration of natural extract formulations was conducted in HEK293TN cells. The cells were treated with BITS-001, -002, -003, -004 and -005 formulations at a seeding density of 5000 cells per well. The formulations were diluted at 1:30, 1:60, 1:120 and 1:240. The cell viability number and optimum ethanol concentration were also determined. The raw data analysis of the relative light units (RLU) showed that the RLU of the test sample was approximately >250,000 (at 1:30 dilution), >600,000 (at 1:60 and 1:120 dilution) and >100,000 (at 1:240 dilution) as compared to the control group (>1,000,000 RLU) (Figure 2a). Furthermore, the cell viability analysis showed <2.5% viability (at 1:30 dilution), >40% (at 1:60 and 1:120 dilution) and ~100% cell viability at 1:240 dilution, as compared to the control group (Figure 2b). Our data showed the maximum non-toxic dose emerged at 1:240 dilution for all the extracts tested, and this was used for all subsequent virus infectivity assays (Figure 2; data shown for BITS-003). The media without cells served as a background control, and media in the presence of vehicle control served as a negative control for maximum viability. This experiment shows that the herb-based formulations BITS-001 to BITS-005 described in the present manuscript are non-toxic to epithelial cells.

### 3.4. Antiviral Activity: Viral Inactivation Assay Results Indicate That Natural Extract Formulation Inactivates the Lentivirus

**Figure 3:**
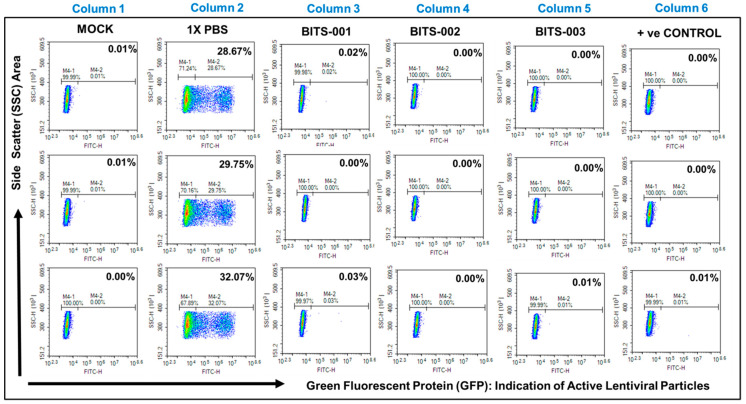
Viral inactivation assay using natural extracts (flow cytometry analysis): Fluorescence Activated Cell Sorting (FACS) analysis of cells transduced using lentiviruses treated with natural extracts at 1:30 dilution for 1 hour. Green Fluorescent Protein (GFP)–positive cells (M4-2) are an indication of biologically active viral particles. A decrease in GFP-positive cells is a direct indication of lentivirus inactivation. A validated chemical (remdesivir) was used as a positive control for viral inactivation.

The lentivirus-transduced cells were treated with natural extract formulation BITS-001, -002, -003, -004 and -005 at 1:30 dilution for 10 min. The BITS-001, -002 and -003 formulations were found to completely inhibit the infectivity of the lentivirus into the HEK293TN cells, as measured by Fluorescence-Activated Cell Sorting (FACS) for green-fluorescent protein (GFP) expressed in cells post successful viral infection (Figure 3). The green-fluorescent protein (GFP)–positive cells indicated biologically active viral particles. Cells were mock-infected using phosphate-buffered saline (PBS), under the experimental conditions of without lentivirus (column 1) or infected with untreated lentivirus negative control (column 2) or virus pre-treated with BITS-001 (column 3), BITS-002 (column 4) or BITS-003 (column 5). No GFP expression could be detected in cells infected with pre-treated virus particles using the formulations described above, depicting complete disruption of the virus envelope and near 100% inhibition of infection in HEK293TN cells (Figure 3).

**Figure 4:**
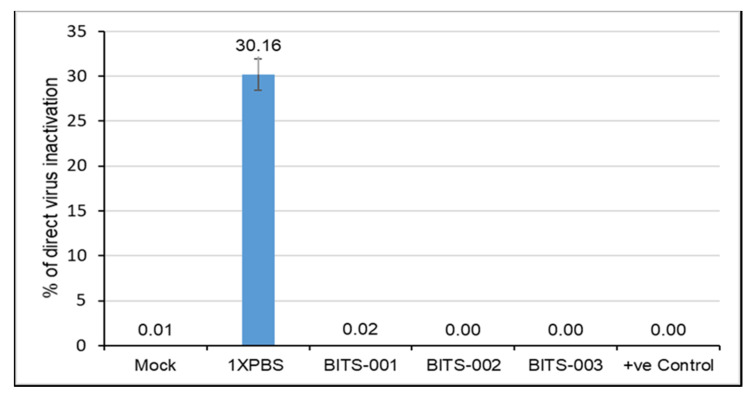
Viral inactivation assay (graphical representation). Bar graph represents quantification of GFP cells at indicated conditions. Virus infectivity observed is 30.16% in 1X PBS-treated samples, whereas natural extract–treated samples (1:30 dilution for 10 min treatment) showed between 0.00% and 0.03% infectivity. Final strength of the formulation in the cell culture medium is at 1:240 dilution.

Furthermore, virus infectivity was observed to be 30.16% in 1X PBS-treated samples, whereas the natural extract–treated samples (1:30 dilution for 10 min treatment) showed between 0.00% and 0.03% infectivity. The final concentration of the formulation in the cell culture medium was at 1:240 dilution, determined to be the maximum nontoxic dilution as per the cytotoxicity assay. The bar graphs clearly show that the lentivirus treated with BITS-001, BITS-002 and BITS-003 showed significantly lower infection and consequently no detectable fluorescence in cells infected with the lentivirus (Figure 4). Figure 4 compares the antiviral potential of various herb-based formulations—BITS-001, BITS-002 and BITS-00—against the lentivirus. This experiment also proves that the herb-based formulation described in the present manuscript possesses potent antiviral properties against enveloped RNA viruses. 

### 3.5. BITS-003 Formulation and Antiviral Activity

**Figure 5:**
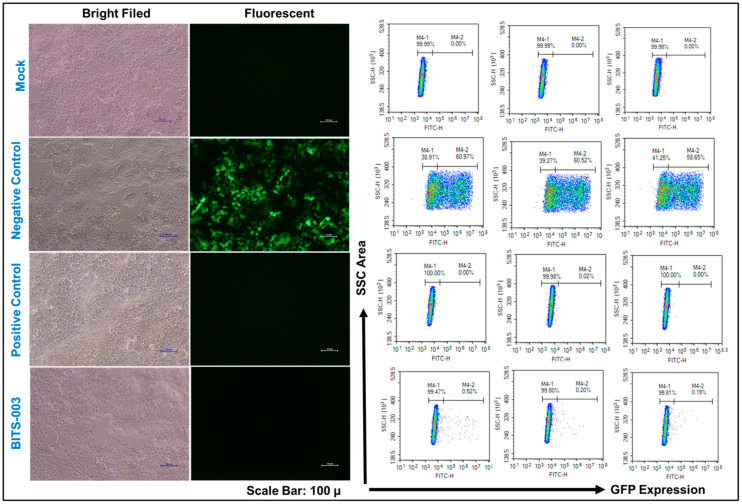
Microscope imaging at 10× magnification (Scale bar: 100 µ) and FACS analysis of cells transduced using lentiviruses treated with BITS-003 at 1:30 dilution for 10 minutes. Decrease in GFP-positive cells is a direct indication of lentivirus inactivation.

The cells transduced using lentiviruses treated with the BITS-003 formulation at 1:30 dilution for 10 min were analyzed by microscope imaging at 10X magnification (Scale bar: 100 µ) and FACS for GFP-positive cells to further confirm the presence of biologically active viral particles (Figure 5). The decrease in GFP-positive cells is a direct indication of lentivirus inactivation. The lentivirus treated with BITS-003 showed no measurable fluorescence, indicating complete inactivation of the lentivirus by the novel herb-based compositions. The antiviral effects of herb-based combination BITS-003 against the lentivirus showed powerful antiviral activity against the lentivirus (Figure 5).

### 3.6. Antibacterial and Antifungal Activity Assay Results—Natural Extract Formulation Inactivates Bacterial and Yeast Cells

**Figure 6:**
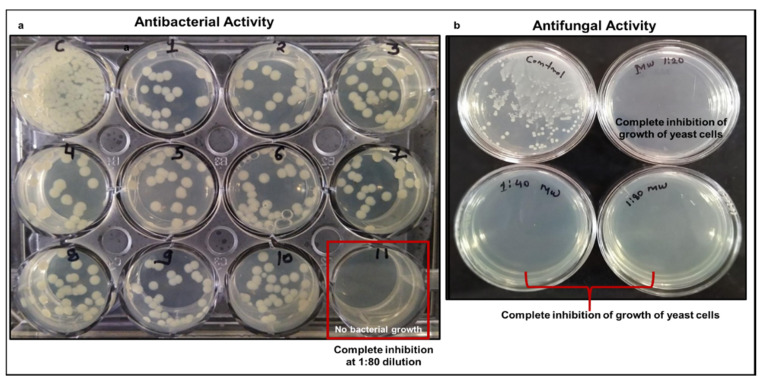
(**a**) The synergistic antibacterial activity of the herb-based formulation. Well C: control; Well 1: *Asparagus rasemosus* at 1:40 dilution; Well 2: *Asparagus rasemosus* at 1:80 dilution; Well 3: *Glycerrhiza glabra* at 1:40 dilution; Well 4: *Glycerrhiza glabra* at 1:80 dilution; Well 5: *Bacopa monnieri* at 1:40 dilution; Well 6: *Bacopa monnieri* at 1:80 dilution; Well 7: *Rosmarinus officinalis* at 1:40 dilution; Well 8: *Rosmarinus officinalis* at 1:80 dilution; Well 9: *Nigella sativa* at 1:40 dilution; Well 10: *Nigella sativa* at 1:80 dilution; Well 11: herbal composition BITS-003 at 1:80 dilution. No bacterial growth was seen at 1:80 dilution in bacterial cells treated with the herb-based formulation. (**b**) The antifungal activity of the herb-based formulation BITS-003 against *Saccharomyces cerevisiae*. The herb-based formulation displayed potent antifungal activity at 1:20, 1:40 and 1:80 dilutions. Control: *Saccharomyces cerevisiae* grown in the absence of herb-based formulation; 1:20 MW: *Saccharomyces cerevisiae* grown in the presence of 1:20 diluted herb-based formulation; 1:40 MW: *Saccharomyces cerevisiae* grown in the presence of 1:40 diluted herb-based formulation; 1:80 MW: *Saccharomyces cerevisiae* grown in the presence of 1:80 diluted herb-based formulation. Complete inhibition of growth was seen for yeast cells treated with the herb-based formulation.

The bacterial cells treated with individual herbal constituents showed reduced bacterial growth as reflected by lower the number of bacterial colonies observed in treated cells (Figure 6a: Well 1−Well 10). The bacterial cells treated with the BITS-003 formulation showed complete inhibition of bacterial growth (Figure 6a: Well 11). The control well shows normal bacterial growth in untreated cells. However, the combination of all herbal extracts in the herb-based formulation showed potent antibacterial activity and completely prevented the growth of *Klebsiella,* a particularly common inhabitant of the oral cavity, suggesting a synergistic antibacterial action of the novel herb-based formulation stronger than the individual herbal extracts alone. Well 11, with herbal composition BITS-003, showed no bacterial growth at 1:80 dilution (Figure 6a). 

Furthermore, we also show that the yeast cells treated with the herb-based formulation displayed reduced growth, as reflected by lower number of yeast colonies observed in the treated cell wells (Figure 6b). The herb-based formulation BITS-003 possesses powerful antifungal activity against yeast *Saccharomyces cerevisiae*, thus making it highly suitable for killing/preventing the growth of fungus in the oral cavity. Complete inhibition of growth of yeast cells treated with the herb-based formulation was observed at 1:20, 1:40 and 1:80 dilutions.

## 4. Discussion

Mouthwashes and gargles are widely used in oral and dental hygiene and are one of the most available solutions to reduce pathogens and microbes. The oral cavity is believed to play an important role in pathogenicity and transmission of viral infection, including SARS-CoV-2. To date, no herbal mouthwash and gargle exists with broad spectrum antiviral, antibacterial and antifungal properties. The recent literature suggests that oral rinses have potent virucidal properties and have the potential to inactivate enveloped viruses such as SARS-Cov-2. Our study shows that several herb-based oral rinses tested during the course of this study were able to reduce the amount of infectious virus by greater than 99.9% within a contact time of just 10 min. In contrast, common saline, as used in a Neti-Pot, had no effect on viral infectivity in our study. Most of the common over-the-counter mouthwashes/gargles tested have demonstrated around a 90% reduction in infectious virus, with varying active drug ingredients and formulations, namely 1.5% Peroxide, 0.07% Cetylpyridium chloride, or alcohol-based eucalyptol, menthol, methyl salicylate, and thymol formulations that historically have claimed numerous antimicrobial properties. All these products claim to only kill the germs that cause bad breath. 

In the context of the pandemic due to SARS-Cov-2 infection, it is critical to develop methods to reduce infection transmission rates. Wearing masks and social distancing can significantly decrease the transmission and spread. Nasal rinses and mouthwashes, which directly impact the major sites of reception and transmission of SARS-Cov-2 and other human coronaviruses, may provide an additional level of protection against the virus. 

Over-the-counter mouthwashes/gargles generally claim to speed the wound healing process, to have antiseptic properties intended to reduce the microbial load in the oral cavity, to prevent gingivitis, and to kill germs that cause bad breath. Mouthwashes are commonly used as oral healthcare products and have traditionally served both cosmetic and therapeutic purposes. To evaluate whether the antimicrobial activity of the formulated mouthwash and gargle developed from plant extracts reduces the percentage of microbial and fungal colonies, the *Klebsiella* strain used to test the antimicrobial activity was found resistant to three antibiotics, namely Cefuroxime, Cefixime and Ceftriaxone and several broad-spectrum antibiotics, namely Amoxicillin, Cefotaxime, Cephalexin and Clindamycin. Still the growth of resistant *Klebsiella* strain was inhibited by the rinse. In addition, there is a large body of literature showing that microbes do not develop resistance to phytoconstituents [38,39,40,41,42].

Oral microbiota is an integral part of oral health and comprises several different bacterial, fungal and protozoan species. Many opportunistic pathogens, such as Candida, can affect the oral mucosal layer, causing symptomatic mucosal infections. Our study demonstrated that the herbal formulation has the ability to inhibit the growth of the yeast cells.

In general, most of the commercially available mouthwashes contain organic solvents or synthetic chemical agents as active ingredients, which makes them unsafe for long-term use. Indeed, all chemical-based mouthwashes come with a warning “not to be ingested”, as the constituent chemicals are known to cause gastric irritation and even ulcers. Several public health experts have proposed the use of mouthwash for controlling the global outbreak of COVID-19, a life-threatening disease caused by SARS-CoV-2. We report here the development of herb-derived phytoconstituent-based mouthwash with strong virucidal properties, inactivating from 2 log 10 (or 99%) to greater than 4 log 10 (or 99.99%) of enveloped viruses as well as antibacterial and antifungal activity. Our studies indicate that phytoconstituent-based natural products containing mouthwash could serve as a safe complement to other healthcare and public health antiviral measures and prevent against infection at both the individual and the community level. 

Like our work, the study by Ao et al., 2021, reported that aqueous extract of natural herb Prunella vulgaris displayed potent inhibitory effects on SCoV-2 SP (including SPG614 mutant) pseudotyped virus (SCoV-2-SP-PVs) mediated infections [43]. Extract of Prunella vulgaris and our formulation share critical phytoconstituents, such as Quercetin, Ursolic acid, Saponins, Caffeic acids, Rosmarinic acid, Luteolin, Kaempferol, Gallic acid, etc. Furthermore, the study by Balkrishna et al., 2021, showed the herbal drug named Coronil effectively inhibited the interaction of ACE-2 with the wild-type S protein (SWT) and also prevented the SARS-CoV-2 S-protein pseudotyped vesicular stomatitis virus (VSVppSARS-2S) mediated cytokine response in these cells by reducing entry of pseudoviruses into host cells [44]. 

In light of this, extracts from a number of medicinal plants and multiple formulations thereof were tested for their ability to inhibit viral infectivity, and a final formulation comprising four extracts was tested for antiviral activity, using a lentiviral system and cultured HEK 293TN cells, as well as antibacterial and antifungal properties.

Physicians, nurses, respiratory therapists, dentists, dental assistants, and others, who need to be in close proximity to the face of another person to do their jobs, are all at enhanced risk, as are also the families of anyone else who may come in contact with an asymptomatic infected person. The emergence of the SARS-CoV-2 pandemic has created an unprecedented healthcare crisis and has led to significant social and economic consequences. The wearing of masks and social distancing can significantly decrease transmission and spread; however, due to circumstances such as medical or dental intervention and personal choice, these practices have not been universally adopted. Additional strategies are required to decrease the transmission from person to person and in the community.

Several possible limitations of this work must be acknowledged. We did not use SARS-CoV-2 in this study as the test virus, as working with this virus required special biosafety level-3 laboratory conditions. Instead, we used high numbers of infectious engineered lentivirus, a common surrogate for enveloped RNA viruses, including SARS-CoV-2. This allowed us to rapidly test a multitude of ingredients and products at varying contact dilutions (data not shown) and optimize the oral wash formulation for mitigation efforts against COVID-19. Future clinical trials and validation will be needed to evaluate the effect of these oral rinses in actual SARS-Cov-2 infected patients.

## 5. Conclusions

Our finding encourages clinical research to recommend herbal mouth rinses as antiviral, antibacterial and antifungal agents. BITS-003 contains immense medicinal values and can be considered as a safe and effective oral hygiene aid. While the world waits for definitive therapies and vaccines to contain and prevent the spread of SARS-CoV-2, additional strategies are required to decrease viral transmission. An antiviral mouthwash, which directly works on the major sites of reception and transmission of human SARS-CoV-2, may provide an additional, highly safe level of protection against this virus. Here, we have shown that drug- and alcohol-free natural product–based herbal formulation BITS-003 has significant virucidal properties with respect to enveloped viruses, including SARS-CoV-2. In vitro studies have confirmed the antiviral, antibacterial and antifungal properties of the herbal mouthwash formulation developed herein, which can reduce the viral, bacterial and fungal load in the oral cavity. The formulation BITS-003 contains a mixture of some of the most revered herbs listed in ancient text of the Indian System of Medicine, namely Ayurveda. These herbs have been used traditionally for centuries; they pose no side effects from regular long-term use and are safe for ingestion, based on their widespread use in obtaining nutraceuticals as health supplements.

## Figures and Tables

**Table 1:** List of selected plant extracts and the final formulations used.

S. No.	Plant Extracts	Formulation (BITS-001, -002, -003, -004, -005)				
		1	2	3	4	5
1	*Oryza Sativa*		√		√	
2	*Asparagus racemosus willd*		√	√	√	√
3	*Solanum tuberosum*		√		√	√
4	*Glycyrrhiza glabra*			√	√	√
5	*Withania somnifera*	√			√	
6	*Zingiber officinale*				√	
7	*Bacopa monnieri*	√	√	√	√	√
8	*Rheum palmatum*					√
9	*Rosmarinus officinalis*					√
10	*Capsicum annuum*		√		√	
11	*Nigella Sativa*			√		
12	*Phaseolus Munga L*	√	√		√	
13	*Trigonella foenum graceum*		√		√	
14	*Ananas Comosus*		√		√	
15	*Colocasia antiquorum Schott*		√		√	
16	*Morus nigra*		√		√	
17	*Utica dioica*		√		√	
	Total No. of extracts used	3	11	4	14	6

**Table 2:** Concentration of total polyphenol, saponin and flavonoid content of compounds in the five formulations.

	BITS 001	BITS 002	BITS 003	BITS 004	BITS 005
Total Polyphenol content	1760.34	1096.33	643.63	1407.48	1259.62
Total Saponin content	16,492.31	14,369.23	19,230.77	15,917.95	14,974.36
Total Flavonoid content	21,900	12,625	2725	10,400	16,075

## Data Availability

Data are available on request from the authors. The data that support the findings of this study are available from the corresponding author upon reasonable request.

## Disclaimer

The contents of this publication and the views expressed in this article reflect the results of research conducted by the authors are the sole responsibility of the author(s) and do not necessarily reflect the views, opinions or policies of Uniformed Services University of the Health Sciences (USUHS), The Henry M. Jackson Foundation for the Advancement of Military Medicine, Inc., the Department of Defense (DoD), or the Departments of the Army, Navy, or Air Force. Mention of trade names, commercial products, or organizations does not imply endorsement by the US Government.
